# Cytomegalovirus-Induced Pericarditis, Pulmonary Embolism, and Transaminitis in an Immunocompetent Patient

**DOI:** 10.7759/cureus.19285

**Published:** 2021-11-05

**Authors:** Waiz Wasey, Navpreet Badesha, Maria Rossi, Caitlin Carter, Samantha Bibee

**Affiliations:** 1 Family and Community Medicine, Southern Illinois University School of Medicine, Springfield, USA; 2 Family Medicine, Southern Illinois University School of Medicine, Springfield, USA; 3 Psychiatry, Southern Illinois University School of Medicine, Springfield, USA

**Keywords:** viral infection, transaminitis, pulmonary embolism, pericarditis, cytomegalovirus

## Abstract

Cytomegalovirus (CMV) is a global virus with a high prevalence that usually manifests in immunocompromised patients, with significant morbidity and mortality. Though fairly common in immunocompetent patients admitted in the intensive care units, the infection is usually subclinical. Healthy individuals either have a subclinical course or exhibit a mild mononucleosis-like syndrome. Due to this, little attention has been given to morbidity and mortality that CMV infection may lead to in immunocompetent patients. We report a case of a 55-year-old immunocompetent female with no significant history who was admitted to our medical service with pericarditis, complicated by a right pulmonary embolism. Infectious workup revealed CMV as the cause for the presenting symptoms.

## Introduction

Cytomegalovirus (CMV) belongs to the genus *Herpesvirales* and chooses its hosts in humans and monkeys. The seroprevalence for CMV worldwide ranges between 60% and 100% [1]. Infection usually presents as congenital infections in the fetus or with an array of presentations in immunocompromised patients [2]. The disease in the immunocompromised patients may manifest through reactivation of latent CMV infection or the acquisition of a new primary infection [3]. The infection may present with a wide array of symptoms that include colitis, myocarditis, pericarditis, retinitis, hepatitis, pneumonitis, or encephalitis [4]. Colitis has been observed as the most common clinical manifestation [3]. In the immunocompetent patients, the CMV infection either runs a subclinical course or presents as a mild viral-like syndrome. Since the presentations in healthy patients are mild, less attention is given to morbidity and mortality in these patients. Also as CMV manifestations mimic an array of other conditions, it poses a diagnostic challenge in immunocompetent individuals [3]. Serological studies are used to screen CMV. The commonly used lab work includes CMV immunoglobulin G (IgG) and CMV immunoglobulin M (IgM) [5].

Thrombosis was considered to be an extremely rare complication of CMV infection with less than 100 reported cases. Recent literature reviews, however, report that at least 5% of hospitalized patients with CMV infection develop deep venous thrombosis (DVT) [6]. The correlation between thrombotic events and CMV infections has been demonstrated in a systematic review including 79 articles with 115 cases [7]. The most common DVTs are those of the lower extremities, followed by splanchnic vein thrombosis [8]. Our report of pulmonary vein thrombosis is a rare complication.

Treatment of CMV with antivirals is reserved for moderate to severe presentations only. Most of the infections are mild and self-limiting and do not need treatment. There are four drugs that are approved for treating CMV. These are ganciclovir, valganciclovir, foscarnet, and cidofovir. The use of these antivirals is limited due to poor bioavailability, potential toxicities, and the development of resistance [9].

We present a healthy female who was admitted with pericarditis and later developed a pulmonary embolism during the course of her hospitalization. The purpose of this case report is to comment on the significant morbidity that may present in healthy individuals infected by CMV.

## Case presentation

**Figure 1:**
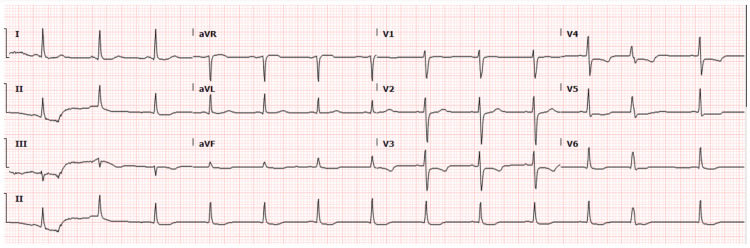
EKG in the ER showing ST wave depression in V3 and V4 and moderate T-wave abnormality.

A 55-year-old female with no significant past medical history, except for anxiety, presented to the emergency room (ER) with chest pain. Initial workup in the ER indicated an abnormal electrocardiogram (EKG). The EKG displayed ST wave depressions in leads V3 and V4 (Figure 1). As a part of chest pain workup, a computed tomography (CT) scan of the chest was done, which was negative for pulmonary embolism (PE).

The initial laboratory evaluations revealed mild leukocytosis with elevated acute phase reactants and mild transaminitis (Table 1).

**Table 1:** Laboratory evaluation in the ER.

Lab test	Value	Reference value
Hemoglobin	12.8 gm/dl	12-16 gm/dl
White blood cells	9.5 k/mm3	3.4-9.4 k/mm3
Platelets	176 k/mm3	140-410 k/mm3
Sodium	138 mmol/L	136-145 mmol/L
Potassium	4.1 mmol/L	3.5-5.1 mmol/L
Creatinine	0.8 mG/dl	0.6-1.3 mG/dl
Troponin	<0.03 ng/ml	<0.03 ng/dl
B-type natriuretic peptide	24 pg/mL	0-80 pg/mL
Alkaline phosphatase	121 IU/L	34-104 IU/L
Aspartate aminotransferase	85 IU/L	13-39 IU/L
Alanine aminotransferase	134 IU/L	7-52 IU/L
C-reactive protein	12.9 mg/L	0-5 mg/L

**Figure 2:**
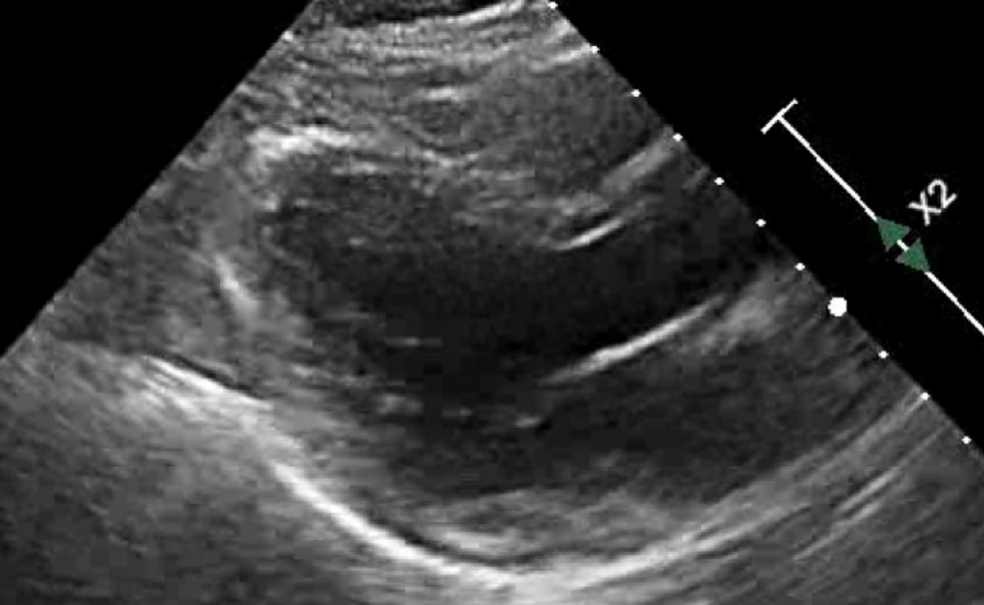
Echocardiogram showing mild pericardial effusion.

With the suspicion of pericarditis, the patient was admitted for further evaluation. A transthoracic echocardiogram (Echo), CT angiogram, and CT cardiac chest were performed during the course of admission. The Echo revealed pericardial effusion (Figure 2) and an ejection fraction of 68% with normal right ventricular function. The CT angiogram and cardiac chest performed on day two of admission revealed a calcium score of 0 with minimal atherosclerotic plaque. Moreover, this time it was positive for a new PE in the right lower lobe.

Given the findings of pericarditis, leukocytosis, and transaminitis, further infectious and inflammatory workup was performed. The workup revealed positive antibodies for CMV infection (Table 2). Autoimmune conditions were also ruled out given the patient developed a PE. A hypercoagulable panel was also ordered.

**Table 2:** Inflammatory and Infectious workup for pericarditis and transaminitis. IgG, immunoglobulin G; IgM, immunoglobulin M; AG, antigen; CMV, cytomegalovirus; PCR, polymerase chain reaction; AB, antibodies.

Lab test	Value	Interpretation
Epstein-Barr virus (EBV) capsid antibody, IgG	>8.0 AI	Positive for antibodies to past infection
EBV capsid antibody, IgM	0.5 AI	Negative
EBV nuclear AG antibody, IgG	0.6 AI	Negative
EBV early AG antibody, IgG	<0.2 AI	Negative
CMV IgG	1.0 AI	Equivocal
CMV IgM	>4.0 AI	Positive for recent or current infection
Human immunodeficiency virus	Not reactive	Negative
Blastomycosis immunodiffusion	Negative	Negative
Histoplasmosis immunodiffusion	Negative	Negative
Histoplasmosis antigen urine	Not detected	Negative
Mononucleosis test	Negative	Negative
Coronavirus 2019 (COVID-19) PCR test	Not detected	Negative
Rheumatoid factor	<10 iU/mL	Negative
Antinuclear antibody	Not detected	Negative
Anticardiolipin AB IgG	<1.6 GPL U/mL	Negative
Anticardiolipin AB IgM	1.0 MPL U/mL	Negative
Factor V Leiden mutation	Not detected	Negative
Prothrombin G20210A gene	No mutation found	Negative
Protein C activity	91%	Negative
Antithrombin III activity	107%	Negative
Protein S activity	52%	Borderline low - can be depressed in acute thrombosis, liver disease, sepsis, and pregnancy

The CMV infection was confirmed with a CMV antigenemia test, which has a sensitivity of 91% and specificity of 98%, and DNA quantification (Table 3).

**Table 3:** CMV DNA quantification studies. CMV, cytomegalovirus.

Lab test	Value	Interpretation
CMV DNA quant. log	4.3 log cpy/mL	Detected
CMV DNA quant. copies	18.205 cpy/mL	Detected

A duplex scan of the lower extremities did not reveal any DVT. The hypercoagulable workup was unremarkable. There were no other risk factors or history of any risk factors in the patient to have explained the PE either. There was no family history of any hypercoagulable disorder. It was concluded that the PE was a result of the underlying CMV infection.

Our patient was hence diagnosed with acute CMV infection leading to pericarditis, complicated by PE. She was started on high-dose aspirin and valganciclovir to treat the active CMV infection and pericarditis. She was also started on rivaroxaban to treat the PE.

Her symptoms improved since being discharged from the hospital and she did not present with any new complications at the one-month follow-up with her primary care physician and continues to follow up with the infectious disease department.

## Discussion

Several reports can be found in the medical literature on manifestations of CMV infections in the immunocompromised population, but very rare data exist for presentations in the immunocompetent population, despite high seroprevalence [1]. It is widely believed that the presentations in those with a good immune system are usually mild and self-limiting. A systematic review performed [2] showed that severe complications of CMV are not as rare as previously believed in immunocompetent patients. In adults, CMV infection can present as fever, pharyngitis, hepatitis, encephalitis, pericarditis, and pneumonia [10]. Severe organ involvement has been reported in the gastrointestinal tract as colitis [11]. In our case, the patient presented with pericarditis and mild transaminitis.

Diagnosing CMV is challenging due to the fact that the clinical suspicion in healthy individuals is low. Also, CMV may present with a wide array of clinical manifestations [3]. And finally, the fact that it may mimic other diseases in its presentations leads to a delay in diagnosing CMV [2]. A variety of laboratory methods are available to diagnose CMV. The most commonly utilized are molecular studies such as CMV-specific IgG and IgM antibodies [12]. We utilized these antibody tests and further confirmed the diagnosis with quantitative and antigenemia tests.

For treating CMV infections, intravenous ganciclovir and oral valganciclovir are preferred as first-line drugs [12]. The clinical decision to treat with antivirals depends on the severity of the illness [9]. Most presentations are mild and self-limiting. The duration of therapy varies on clinical and laboratory response. Weekly quantitative, as well as antigenemia tests, are required to predict positive responses. We discharged our patient on oral valganciclovir. Other treatment options include foscarnet, cidofovir, and a novel oral prodrug called brincidofovir [12]. Once viral suppression is seen, the antivirals need to be continued for two more weeks prior to discontinuation. This is to prevent disease relapse.

Since the 1980s, thrombosis associated with CMV infections has been reported. The association between vascular thrombotic events and CMV infections has been demonstrated in a systematic review including 79 articles of 115 cases [7]. The incidences of thrombosis are rare and in hospitalized patients, and the incidence was seen to be 6.4% [13]. Lower limb thrombosis was the most prevalent, followed by splanchnic vein thrombosis [8]. Very few cases of PE have been reported. It is hypothesized that the mechanism of thrombosis in CMV infection is through direct damage to endothelial cells, increased levels of hemostatic indicators, and induction of smooth muscle proliferation [14]. Our patient was diagnosed with PE incidentally through a CT angiogram on day two of hospitalization. Her initial CT scan in the ER did not reveal any PE on admission. PE or DVTs are usually treated with anticoagulants [15].

Our patient was diagnosed with acute CMV infection leading to pericarditis, complicated by right lower lobe pulmonary embolism. She was sent home on oral valganciclovir and rivaroxaban.

## Conclusions

In conclusion, CMV infections are perceived to be rare in immunocompetent patients but are more common than initially thought. They present with a wide array of presentations, which makes diagnosing it a challenge. Our report aims in making clinicians more aware of acute CMV infections in healthy individuals, especially with presentations of pericarditis and pulmonary embolism.
